# Intertemporal Choice as Discounted Value Accumulation

**DOI:** 10.1371/journal.pone.0090138

**Published:** 2014-02-28

**Authors:** Christian A. Rodriguez, Brandon M. Turner, Samuel M. McClure

**Affiliations:** 1 Department of Psychology, Stanford University, Stanford, California, United States of America; Brain and Spine Institute (ICM), France

## Abstract

Two separate cognitive processes are involved in choosing between rewards available at different points in time. The first is temporal discounting, which consists of combining information about the size and delay of prospective rewards to represent subjective values. The second involves a comparison of available rewards to enable an eventual choice on the basis of these subjective values. While several mathematical models of temporal discounting have been developed, the reward selection process has been largely unexplored. To address this limitation, we evaluated the applicability of the Linear Ballistic Accumulator (LBA) model as a theory of the selection process in intertemporal choice. The LBA model formalizes the selection process as a sequential sampling algorithm in which information about different choice options is integrated until a decision criterion is reached. We compared several versions of the LBA model to demonstrate that choice outcomes and response times in intertemporal choice are well captured by the LBA process. The relationship between choice outcomes and response times that derives from the LBA model cannot be explained by temporal discounting alone. Moreover, the drift rates that drive evidence accumulation in the best-fitting LBA model are related to independently estimated subjective values derived from various temporal discounting models. These findings provide a quantitative framework for predicting dynamics of choice-related activity during the reward selection process in intertemporal choice and link intertemporal choice to other classes of decisions in which the LBA model has been applied.

## Introduction

In order to choose between rewards available at different points in time it is often necessary to evaluate the tradeoff between the size of potential rewards and the corresponding delays until their receipt. For example, deciding whether to save or spend a certain amount of money requires determining whether ensuring greater future wealth is worth delaying the pleasure of spending and consuming now. When engaged in this form of decision making, a class of decisions known as intertemporal choice, humans and other species discount the value of rewards in proportion to the delay at which they are available. Moreover, the behavior observed in intertemporal choice experiments reveals preferences consistent with a steep reduction in the value of rewards delayed from the present moment but more modest discounting of rewards delayed from future time points [1]. This property is particularly evident as a greater reluctance to forego immediate for delayed rewards compared with when both outcomes are delayed, a tendency that manifests itself in impulsivity and a predilection for procrastination. Several mathematical models have been shown to account for this pattern of delay discounting [2]. However, subjective valuation is only one of the cognitive processes involved in intertemporal choice behavior [3], [4].

In addition to representing the value of delayed rewards, intertemporal choices require comparing alternatives and selecting among them. One proposal for how delayed rewards might be compared and selected is through a process of sequential sampling of discounted values [3]. Similar processes are commonly assumed to underlie perceptual judgments based on sensory evidence [5]. This hypothesis suggests that there exists a direct connection between choices made on the basis of discounted values and other choices which have been argued to derive from sequential sampling processes. However, the hypothesis that a sequential sampling process underlies intertemporal decision-making has not been empirically tested. Therefore, our primary goal is to determine whether intertemporal choice behavior can be explained by a sequential sampling process based on discounted value.

There are several computational models that employ sequential sampling mechanisms to explain choice behavior (cf. [6]–[8]). A major accomplishment of all of these models is their ability to provide a process-level account of how experimental manipulations such as time pressure and stimulus ambiguity simultaneously affect response times (RT) and error rates. While many of these models might be able to explain intertemporal choice behavior, we used the Linear Ballistic Accumulator (LBA) model [8] in our analyses. The LBA model incorporates the fundamental features of all sequential sampling models, including trial-to-trial variability in the rate of evidence accumulation, a decision criterion, and constants to account for perception and motor execution times. The major advantage of the LBA model is its analytical tractability, which facilitates testing several versions of the model to determine which combination of parameters best accounts for intertemporal choice behavior. We show that the LBA model provides an excellent description of the relationship between choice outcomes and RT and that best-fitting model parameters can be directly related to subjective values.

## Materials and Methods

### Subjects

Fifty healthy adults participated in this study (28 females, ages 19–46 years, mean 24.36 years). All subjects gave written informed consent. Stanford University's Institutional Review Board approved the study. One subject was excluded because the behavior did not allow us to estimate reliable temporal discounting parameters. Another three subjects were excluded because of data collection problems. Data from a total of forty-six subjects were analyzed (28 females, ages 19–46 years, mean 24.26 years).

### Temporal discounting model and task design

The experiments were conducted over two sessions. The purpose of the first session was to estimate each individual's discount rate using a hyperbolic discounting model. For half of our subjects (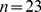) the second session consisted of an electroencephalography (EEG) experiment. For the other half the second session consisted of a functional magnetic resonance imaging (fMRI) experiment. The analyses reported below were obtained from the behavior observed during these EEG and fMRI sessions.

We assumed that the subjective value of delayed rewards was discounted according to 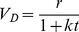
(1)where 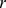 is the magnitude of a reward offered at delay 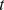. The individually-determined parameter 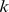 is the discount factor [9]. While subjects completed the first session, we used a stair-stepping procedure to approximate 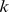. All choices required participants to select between a delayed reward (of amount 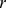 available at delay 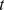) and a fixed immediate reward of $10. For any choice, indifference between the immediate and delayed options implies a discount rate of 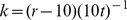. We refer to this implied equivalence point as 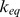; our procedure amounted to varying 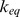 systematically until indifference was reached. Specifically, we began with 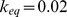. If the delayed offer was chosen, 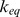 was decreased by a step size of 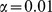 for the next trial. Otherwise, 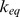 increased by the same amount. At every second choice reversal, occurring within five consecutive trials, the step size was reduced by 5%. A total of 60 trials were completed. We placed no limits on the time subjects could take to respond, and presented both offers on the screen, as “$10 now” on the left side, and “$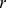 in 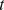 days” on the right.

Critically, our use of the hyperbolic discounting model to summarize behavior in this first experimental session had no bearing on the modeling results that follow. We used the hyperbolic model because it provided a good fit to behavior with a single parameter (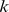) summarizing preferences. Fits of this model were used solely to generate choices for the second experimental session. Alternative delay discounting functions that may or may not provide better fits to behavior would have a subtle impact on the choice set (dollar amounts of choice options) for the second study, but no impact on the model fitting that is the primary aim of the current study.

After completing the first session, we fit a softmax decision function to participants' choices. Intuitively, this procedure allowed us to determine how consistently participants selected the option with greater subjective value. Practically, we fit the softmax to better equate choices during the second session, across participants. In particular, our aim was to equate the relative impact of delayed rewards, across subjects, with respect to actual choice outcomes (i.e. the likelihood of selecting the delayed option). Best fitting softmax functions were estimated by maximizing the likelihood of observed choices. We assumed that the likelihood of selecting a delayed reward (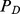) was given by 
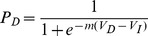
(2)where 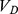 is given by Equation 1, 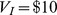 (i.e., the fixed-value of the immediate reward also given by the right side of Equation 1) and 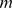 describes a subject's sensitivity to changes in 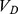.

**Figure 1:**
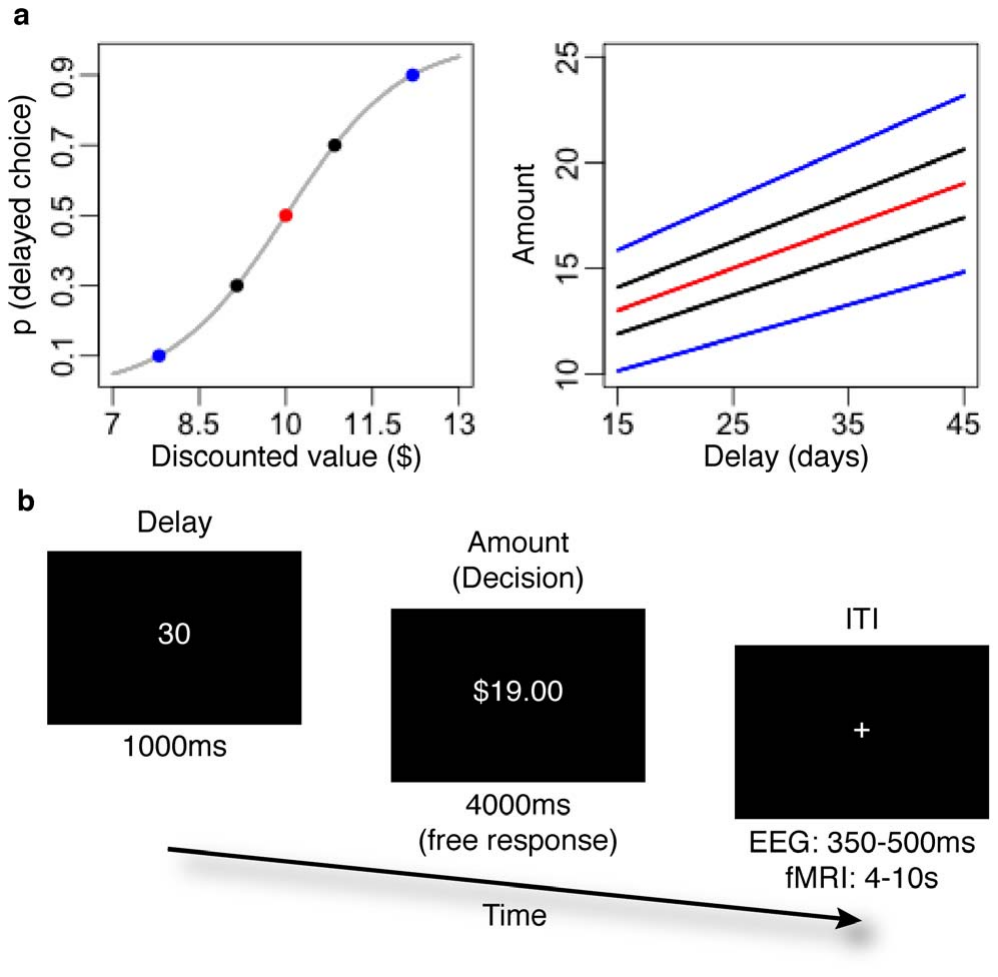
Experimental design. (a) Delayed reward offers corresponded with one of five different levels of discounted value. Each level of discounted value corresponded to one of five probabilities of choosing the delayed reward: 0.1, 0.3, 0.5, 0.7 or 0.9. (b) Every delay could be combined with any of five different amounts to yield a different discounted value and probability of choosing the delayed reward. (c) Delay and amount information was presented sequentially. Delays were presented first for 1000 ms. Amounts were presented second, replacing the presentation of the delay and remaining on the screen for a maximum of 4000 ms. After every trial, a fixation cross was presented on the center of the screen for a randomly chosen inter-trial-interval in the order of hundreds of milliseconds during the EEG experiment and several seconds during the fMRI experiment.

We used individually determined values of 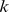 and 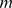 to generate choices for the second session. At every trial, 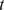 was randomly selected from a range of 30–45 days. We then calculated and offered an amount 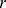 that would give 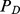 of 0.1, 0.3, 0.5, 0.7, or 0.9 (Figure 1a-b). The EEG group completed 30 trials at every 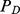 level, except at 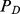 = 0.5, for which they completed 60 trials. The fMRI group completed 40 trials at every 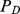 level, except at 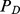 = 0.5, for which they completed 80 trials. Non-uniform trial distributions as a function of 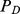 were introduced to allow us to study the effects of choice difficulty on EEG and fMRI measures, with equal numbers of trials at each difficulty level. We report the results of these analyses elsewhere. Trial types were randomized and counterbalanced over two blocks for the EEG group and over four blocks for the fMRI group. We also counterbalanced the mapping between choices and button presses for every subject. During the first half of the second session, approximately half of subjects (13 in EEG, 11 in fMRI) indicated choices of the delayed reward by pressing a button with their left index finger and immediate choices by pressing a different button with their right index finger. The other subjects indicated their choices by the inverse left-right mapping. All subjects switched the initial response mapping during the second half of the session.

To ensure reliable neural measures, we used a sequential presentation of delay and amount during the second session (Figure 1c). During pilot studies we found that a simultaneous presentation of delay and amount caused participants to sequentially fixate the information, producing excessive EEG artifacts. Having the information presented sequentially allowed subjects to maintain central fixation during the task, avoiding these artifacts. As we show below, this sequential presentation of delayed reward information had no adverse effects on behavior. We maintained the same sequential presentation during the fMRI study for the purpose of facilitating direct comparisons and pooling of behavioral data. We report RT as measured from the onset of the decision period, 1000 ms into the trial. The duration of the decision period was fixed at 4000 ms. When subjects made choices in less than 4000 ms the amount information disappeared and the screen remained blank until 4000 ms elapsed. Trial length was thus fixed at 5000 ms. We discarded any trial in which a response was made in less than 200 ms or fell outside of the decision period. To optimize experimental time and separability of neural signals across trials for both groups, we introduced a long inter-trial-interval for the fMRI group (between 4–10 s), whereas the inter-trial-interval was shorter for the EEG group (100–350 ms). In exchange for participation subjects received $10 cash and an additional amount, determined by their choice in a randomly selected trial, taken from either the first or second sessions.

### Model specification and fitting

**Figure 2:**
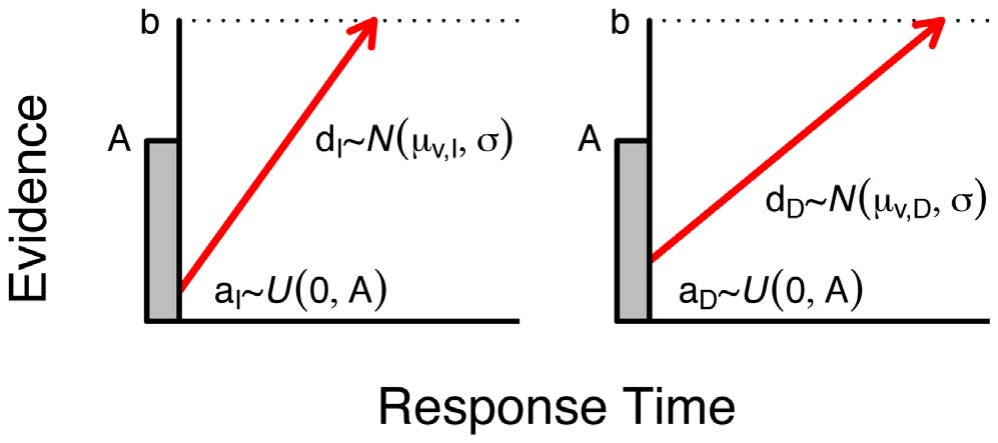
Illustrative diagram of the Linear Ballistic Accumulator model for intertemporal choice, where each response option is represented as a separate accumulator. Following the presentation of a stimulus and some non-decision time 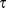, information accumulates ballistically for each alternative. A decision is made that coincides with the accumulator that reaches the threshold 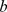 first. The model assumes trial-to-trial variation in both starting point and drift rate.



Figure 2 provides an illustrative diagram of our LBA model of intertemporal choice. To provide a formal description of the model, we denote the RT on the 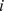
th trial for the 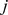
th subject in the 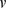
th value condition as 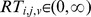, and the corresponding choice as 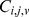 where 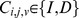. 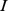 and 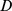 are the immediate and delayed rewards respectively. The model assumes that evidence for 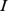 and 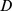 is accumulated independently in separate accumulators. Both accumulators begin with some choice bias, which is provided as independent amounts of starting point evidence 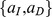, sampled from a common uniform distribution 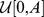. Evidence then increases through time at rates 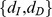, which are sampled from independent normal distributions with means 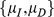. Mean accumulation rates vary across value conditions, but the standard deviation 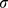 is the same for 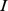 and 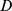. Therefore, 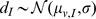 and 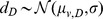. Each accumulator gathers evidence until either one reaches a response threshold 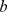. The observed RT is the sum of the decision time, plus some extra time 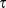, which accounts for non-comparison and selection processes, such as temporal discounting and motor execution. Letting 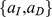  =  **a** and 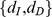  =  **d**, the RT in any given trial is given by

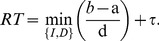
(3)The model provides a closed-form and joint account of RT and choice probability across value conditions by specifying “defective” probability density functions (PDF) for 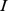 and 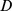 in terms of the parameters just described. These defective PDFs give the probabilities of each accumulator reaching the bound at time 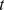. For our best fitting model, the full PDFs are given by 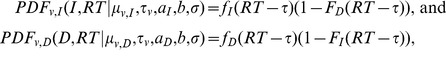
(4)where 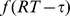 and 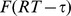 are the PDF and cumulative density functions of each accumulator (see [8] for details).

We estimated LBA model parameters using a hierarchical Bayesian procedure. This procedure offers two advantages over conventional maximum likelihood methods, providing measures of uncertainty for every parameter estimate and allowing the sharing of information across subjects (e.g., [10], [11]), which improves fitting accuracy[12]–[14]. We assume that the data for each subject is characterized by an individual set of LBA model parameters 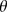, and that these subject-specific parameters are constrained by a set of group-level parameters *ϕ*, which characterize the central tendency and dispersion of 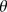 across subjects. The procedure first samples the posterior distributions for every subjects' 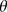 and uses these estimates to derive the posterior distribution of *ϕ*. On every subsequent iteration, the posterior estimates of *ϕ* are used to constrain the sampling of possible values of 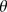 for every subject. We specified mildly informative priors for 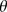, based on empirical evidence from previous fits of the LBA model using the hierarchical Bayesian procedure [15]. For *ϕ*, we specified a conjugate relationship between prior and posterior (see, e.g., [16]). Assuming a conjugate relationship at the group-level allowed us to derive exact conditional posterior distributions, so that we could perform the estimation of all of the parameters simultaneously, based on a single sample of subject-level parameters. The joint posterior distribution estimated by this procedure is given by:
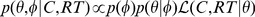
(5)where 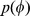 is the prior distribution for 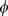, 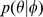 is the prior distribution for 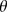 given *ϕ*, and 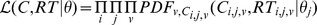
is the likelihood function of the data under the LBA model (given by Equation 4).

To satisfy scaling conditions, we imposed a constraint such that the drift rates sum to one (i.e., 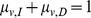). Consequently, it is sufficient to only estimate the drift rate for the delayed reward. For the subject-specific parameters, we first transformed the parameters so that they had continuous, infinite support (i.e., can take on any real value). Thus, for parameters bounded by zero, we applied a log transformation, whereas for the drift rates – which were bounded by zero and one – we used a logit transformation. Following these transformations, we specified the following priors for 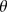: 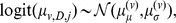


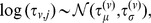


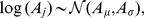


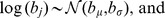


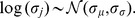



To obtain the desired conjugate relationship between prior and posterior at the level of *ϕ*, we specified the following priors for the group-level means: 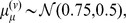


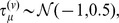


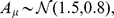


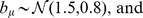


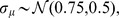
and the following priors for the group-level standard deviations, 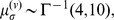


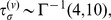


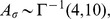


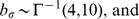


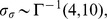
where 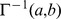 denotes the inverse gamma distribution with shape parameter 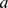, and scale parameter 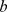. This particular choice of 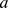 and 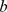 for the priors produces a skewed distribution with an approximate 95% credible set of (1.14, 9.05), and an expected value of 3.32. These choices reflect our *a priori* beliefs: we did not expect the between-subject variability to be less that 1, and felt that larger values would become increasingly less likely to account for these data.

While our prior selections were informed by other similar modeling applications (see, e.g., [15]), we remained conservative in our choices to avoid undue parameter constraint, because our experimental task was considerably different from prior research using the hierarchical version of the LBA model.

We used Gibbs sampling to estimate parameters at the group-level[16], and differential evolution with Markov chain Monte Carlo to estimate parameters at the subject-level (DE-MCMC;[15], [17]). For the subject level estimates, we used 24 chains and obtained 5,000 samples after a burn-in period of 5,000 samples. We then thinned the chains to reduce autocorrelation by retaining every fourth sample. Thus, our estimates of the joint posterior distribution of LBA model parameters are based on 30,000 samples. The burn-in period allowed us to converge quickly to the high-density regions of the posterior distribution, while the rest of the samples allowed us to improve the reliability of the estimates.

To find the optimal number of parameters needed to account for intertemporal choice behavior, we tested a variety of model variants where different sets of parameters were assumed to vary across value conditions. We fit a total of eight variants, following a model building approach based on the Bayesian predictive information criterion (BPIC; [18]). Table 1 shows the model variants we fit (left column) with the particular constraints that were imposed (right column) along with the resulting BPIC values obtained (middle column). We started with the simplest possible model and added parameters only if they improved model fits on the basis of BPIC. The most basic model (M1) only allowed the mean drift rates 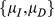 to vary across value conditions. Another four models freed each of the remaining parameters (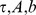 and 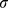), independently, across value conditions. Because the model that freed 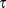 (M2) was superior to M1, we considered three additional models that freed 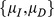 and 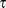, together with each of the remaining parameters independently. None of these three models improved fits, indicating that no additional parameter combinations needed to be tested. We did not consider any models that freed parameters other than 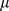 between 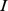 and 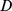 because we found no *a priori* justification for them.

**Table 1:** Mean Bayesian predictive information criterion fit statistics for each model variant we tested (standard deviations of the BPIC values computed across chains appear in parentheses).

Model	BPIC (std. dev.)	Constraint
M1	20101.37 (19.02)	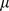
**M2**	**20090.73 (26.91)**	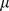 , 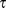
M3	20168.13 (71.25)	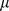 , 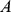
M4	20197.20 (58.93)	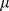 , 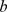
M5	20135.33 (46.86)	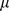 , 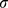
M6	20138.64 (98.01)	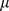 , 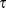 , 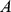
M7	20111.70 (44.94)	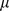 , 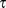 , 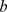
M8	20153.45 (28.34)	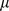 , 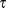 , 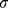

For each model, the third column indicates the set of parameters assumed to vary across value conditions.

## Results

### Model fits

**Figure 3:**
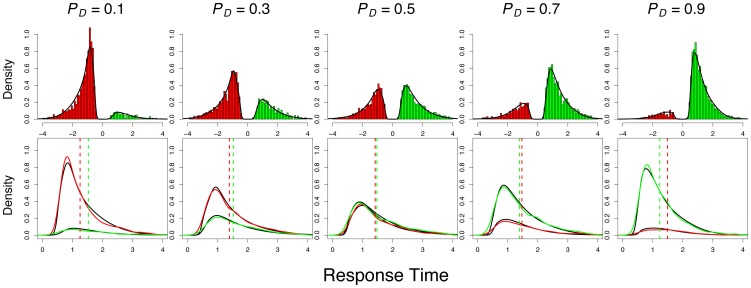
A comparison of model fits to empirical data. The top row shows the aggregated posterior predictive distribution (densities) overlaid on the aggregated empirical data (histograms). The response time distribution for the immediate reward is plotted on the left (i.e., with a negative axis; red), whereas the delayed reward is plotted on the right (green). The choice probability can be inferred by comparing the relative heights of the two distributions. The bottom row shows the same distributions as overlapping density functions with corresponding colors. The model fits are shown as black densities. The median response times for the empirical data are shown as the dashed vertical lines with corresponding colors.

Table 1 shows BPIC results for all the models tested. The best overall model, albeit by a small margin, was M2, which allowed mean drift rates (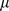) and non-decision times (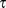) to vary across experimental conditions. Figure 3 shows the quality of the fits obtained with this model. The match between the data and the model predictions is clear in each of the defective PDFs and histograms shown on the top row. These fits speak to the LBA model's ability to simultaneously account for observed RT distributions and choice probabilities during intertemporal choice.

The bottom row of Figure 3 shows the model fits with the RT distributions for both accumulators on the same axis to better illustrate the relationship between choice probability and RT. As net value (i.e. 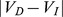) increases, choices for the reward of less subjective value are slower relative to choices for the reward of greater value. This finding is illustrated by the increased separation of RT medians as the probability of choosing the delayed reward deviates further from 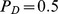 (Figure 3). We confirmed the reliability of this pattern in the data by analyzing RT medians for choices that were consistent versus inconsistent with estimated subjective values. Specifically, we performed a rank-test on RT medians for consistent and inconsistent choices for all value conditions for which 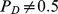 and confirmed that inconsistent responses were slower relative than consistent responses in all conditions where 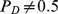 (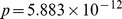). A similar relationship between RT and choice probability is commonly observed during perceptual decision making under stressed accuracy conditions. As choice probabilities deviate from 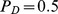, the means of the drift rate distributions (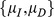) grow further apart (cf. [19], [20]). Recall that 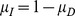. However, subjects maintain an elevated accumulation bound (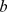) relative to the starting points (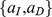). As a result, choices for the reward of less subjective value only occur in the improbable trials where the drift rate for the highest valued reward is unusually low, the drift rate for the lowest valued reward is unusually high, and subjects require more accumulated information before a decision can be made. If the starting points were large relative to the decision bound we would observe the opposite interdependence of RT and choice probabilities. Inconsistent choices would be faster than consistent choices, because fast errors occur when the initial choice bias drives the accumulation close to the decision bound before much evidence influences the decision. This value accumulation mechanism can explain why our model fitting results indicated that variability in 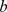 or 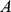 was not required to provide a good fit for these data (i.e., M1 and M2 performed better than M3, M4, M6, and M7).

### Non-decision time

**Figure 4:**
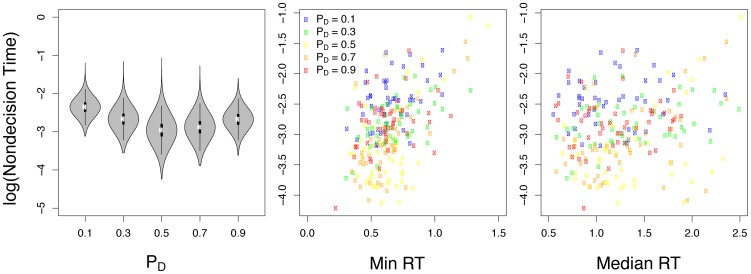
Relationships between model parameters, choice probability, and RT statistics. The left panel shows the estimated group level non-decision time parameter for each value condition. The middle and right panels show the maximum a posteriori (MAP) estimate for each subject's non-decision time parameter against their minimum and median response time, respectively.

The best fitting model, M2, specifies a total of 13 subject-specific parameters, four more than the next best, and simplest model, M1. The four additional parameters modeled differences in non-decision time (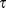) by value condition (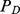). To evaluate whether there was indeed systematic variance in non-decision time, we first inspected group-level estimates of 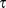, shown in the left panel of Figure 4. These parameter estimates showed a positive quadratic pattern centered at 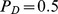. To test the quadratic relationship between 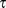 and value, we performed a mixed-effects regression analysis with the nlme package in R (Jose Pinheiro et al., 2013), specifying subjects as random effects, and the regressor 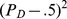 as a predictor of subject-specific maximum *a posteriori* (MAP) estimates of 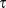. The results corroborated a positive quadratic relationship between 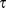 estimates and value (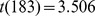, 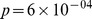), suggesting that there is an increase in valuation and/or motor-execution times as net value increases.

In the LBA model, 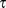 functions as an offset term that captures differences in condition-wise RT that are not captured by the other parameters. The obvious empirical statistics related to average RT differences are condition-wise median and minimum RT. We therefore next tested whether (1) 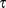 estimates were related to either median or minimum RT, and (2) whether minimum and/or median RT differed by value condition as suggested by the positive quadratic relationship between 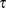 estimates and value.

The middle and right panels of Figure 4 plot subject-specific MAP estimates of 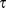 against minimum and median RT, respectively. We conducted two mixed-effects regressions (using subjects as random effects) to determine whether 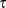 estimates were related to minimum or median RT at each value condition. As hypothesized, 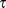 estimates showed a significant linear relationship with minimum RT (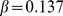, 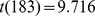, 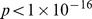) and also a significant linear relationship with median RT (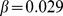, 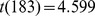, 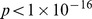).

Given these results, we next sought to determine whether RT differed across value conditions in the same manner as did estimates of 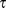. To test this hypothesis, we ran two additional mixed-effects regressions using the quadratic regressor 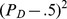 as a predictor of minimum and median RT (with subjects again as random effects). Recall that 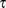 estimates showed a *positive* quadratic relationship with value. This relationship with value was not evident in analyses of minimum or median RT. Specifically, minimum RT did not show a significant quadratic relationship (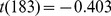, 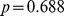), and median RT showed a significant *negative* relationship with value (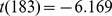, 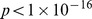). We conclude from these results that neither minimum nor median RT alone can explain the positive quadratic relationship between 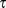 and value. Taken together, our results suggest that the additional degrees of freedom in M2 allowed the model to capture within-subject changes in minimum RT and residual variance of median RT across value conditions.

### Drift rates and value

**Figure 5:**
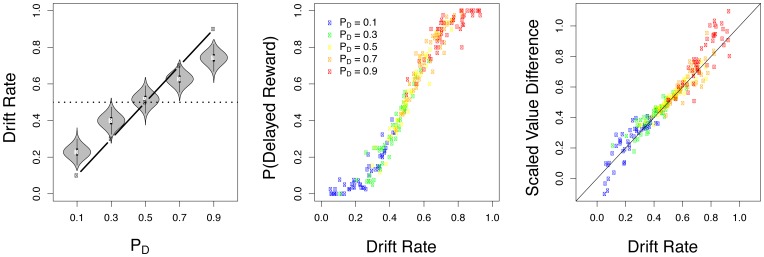
Relationships between model parameters, choice probability, and discounted value. The left panel shows the estimated group level drift rate for each value condition. The middle panel shows the maximum a posteriori (MAP) estimate for each subject's drift rate against observed choice probabilities for the delayed reward (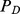). The right panel shows the MAP estimate as a function of subject-specific discounted values for the delayed reward (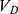).

To obtain a more precise characterization of M2 as a mechanistic theory of discounted value accumulation, we examined the relationship between independently estimated accumulation rates and discounted values. We first tested whether there were systematic differences in group-level estimates of 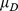 as a function of 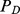. Group-level means of 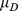 increased as a function of 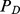. Specifically, we ran a mixed-effects regression of subject-specific MAP estimates of 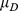 on 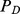 (using subjects as random-effects). This test revealed a significant positive linear relationship (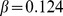, 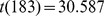, 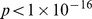; Figure 5, left plot).

Next, we tested for a relationship between observed choice probabilities for the delayed reward and MAP estimates of 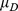 and 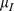 at the level of individual subjects. Specifically, we hypothesized that drift rates (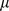) should be related to subjective value through a linear transform, with a slope parameter to account for differences in scale (i.e. 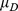 and 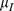 are restricted to be between 0 and 1 but 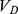 and 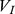 are in dollars with a mean of $10) and an offset parameter to account for differences in drift rate and value means. We further reasoned that if drift rates were directly related to discounted subjective value then drift rates ought to be related to choice probability in the same way that differences in value are related to choice probability. Based on fits of the hyperbolic temporal discounting model (Equation 1) to choice outcomes, we already knew that a sigmoidal relationship (Equation 2) existed between subjective value (i.e. 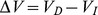) and choice probabilities (i.e. 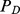). If modeled drift rates had the same relationship then we would expect a similar relationship between 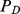, 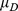, and 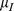. However, 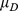 and 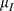 were not independent in our model specification. They were restricted such that 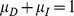. Thus, the difference in drift rates, 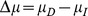 reduces to a linear transformation of 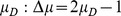. We therefore tested whether a sigmoidal relationship exists between subject- and condition-specific 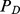 and a linear transform of 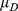: 
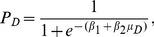
(6)where 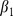 and 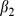 are subject specific parameters.

We tested for evidence to support Equation 6 in two ways. First, we performed a mixed-effects logistic regression using 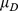 to predict 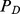, with subjects as random effects. This analysis revealed a significant fit (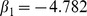, 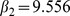, 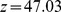, 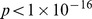). The sigmoidal relationship is also clearly evident in the center plot of Figure 5 which plots 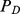 against 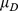. Next, we tested whether the relationship between 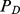 and 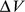 (i.e., Equation 2) was directly related to the relationship between 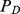 and 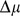 (i.e., Equation 6). If so, then the logistic function in both analyses should be equivalent and the following relationship should hold: 
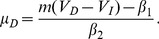
(7)We estimated all of the parameters in Equation 7 from separate logistic regression analyses. Namely, 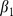 and 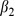 were obtained from fitting Equation 6, 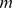 derived from fitting Equation 2, and 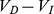 was obtained from best fits of Equation 1, all independently for every subject. In a group-level analysis, we used a mixed-effects regression with subjects as random effect and the right side of Equation 7 as the predictor. This analysis revealed a highly significant slope near unity (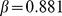, 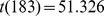, 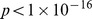). Together, these analyses indicated that there was a strong and direct relationship between drift rates and discounted value. Parameter estimates derived from fitting the LBA model to behavior therefore provided an independent means of estimating subjective values. Moreover, subjective values estimated from the LBA model corresponded closely with values estimated using a hyperbolic discounting model.

### Generalizability of the relationship between drift rates and value

The previous analysis showed that a relationship existed between drift rates derived from LBA model fits and subjective value calculated based on a hyperbolic discount function. Of course, subjective value may actually be determined in a manner that differs in functional form from the hyperbolic equation (cf. [2]). Indeed, numerous functions have been proposed to account for delay discounting. In this final section, we aimed to show that drift rates derived from the LBA model are related to subjective value more generally; that is, that the relationship between drift rates and subjective value does not strictly depend on capturing subjective value using the hyperbolic discount function. To do so, we first fitted two additional discounting models to individual subjects' choices, substituting the right side of Equation 1 with exponential and “quasi-hyperbolic” value functions. For the exponential discounting function, we assumed 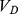 to be given by:
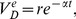
(8)where 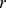 is the delayed reward amount, 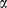 is the discount rate, and 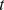 is the delay. Similarly, for the quasi-hyperbolic discounting function, we assumed 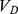 to be given by:
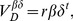
(9)where 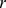 is again the delayed reward amount, 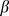 is 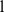 when there is no delay or some fixed value between 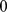 and 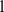 when there is a delay, 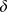 is between 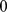 and 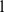, and 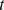 is the delay (always greater than zero).

We then obtained estimates of 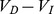 using Equation 8 and Equation 9, as well as two independent estimates of 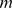, one for each discounting function, from Equation2, for every subject. Next, we ran mixed-effects regression analyses with subjects as random effect and the right side of Equation 7 as predictors of subject-specific drift rate estimates. The analysis using 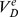 revealed a significant slope near unity (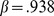, 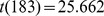, 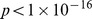) and the analysis using 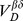 also revealed a significant positive slope (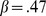, 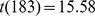, 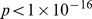). We therefore conclude that drift rates are related to subjective value independent of the specific functional form assumed for delay discounting.

## Discussion

We have shown that intertemporal choice behavior is consistent with a process of discounted value accumulation instantiated by the LBA model. Our findings support the broader hypothesis that selecting among delayed rewards can be explained by a sequential sampling process that corresponds closely with mechanisms known to predict other types of choices (cf. [3]). Thus, perceptual and value-based decision making may depend on similar comparison and selection processes. It is interesting to speculate on whether this similarity reflects a direct correspondence between the cognitive and neural processes that support selection across diverse domains or whether there is simply a common motif for action selection used in separate choice domains.

The LBA model we employed here has been used to explain neural activity during perceptual decision making (cf. [20]–[22]). Furthermore, sequential sampling processes such as that implemented by the LBA model provide a direct link between neural dynamics and decision making behavior. For example, evidence about visual motion is believed to be integrated in the lateral intraparietal (LIP) area, resulting in a progressive increase in LIP neuron firing rates that reflect the accumulation of sensory evidence and predict choice outcomes and response times [23], [24]. Our results represent a first step in extending such findings from perceptual decision making tasks to generate quantitative predictions about discounted value accumulation in intertemporal choice. Moreover, our hierarchical LBA model fitting method might be particularly advantageous for studying the neural mechanisms of value accumulation when used in combination with the “joint modeling framework”, which was designed to simultaneously explain neuroimaging and choice data [25], [26]. Using this framework, [25] have shown that it is possible to link neural and behavioral measures in a way that maps the mechanisms assumed by cognitive models directly to neural function. This approach allows for the specification of *a priori* predictions for how neural mechanisms should influence the modeled cognitive processes that presumably best explain behavior, providing a basis for hypothesis tests that are simultaneously informed by neural data, model parameters, and behavior.

Our results revealed a relationship between response time and choice probability, such that low probability choices are associated with increased response time. Similar results have been observed in previous studies using accumulation models to account for behavior in risk preference[27]–[29] and simple choice tasks [30]–[34]. Our observation that the LBA model can accommodate the relationship between response times and choice probability during intertemporal choice is thus consistent with previous findings and suggests that the LBA model might also be useful in accounting for behavior in other value-based decision domains.

Our best-fitting model included variability in drift rates and non-decision times across value conditions. This result violated our *a priori* expectation that drift rate variability across value conditions would be sufficient to account for our behavioral manipulation. Moreover, our results indicate that the model containing non-decision time variability performed only slightly better than the simplest model which was consistent with our theoretical expectation. Thus, from a purely theoretical standpoint, we favor the simplest model. However, for methodological consistency and empirical validity, we supported and analyzed the fits obtained from the best-fitting model. The BPIC statistic provides a measure of model quality that penalizes for the total number of parameters in the model [18]. Relying on the BPIC statistic we corroborated our prediction that very few parameters needed to vary across conditions, but also found that the best model was not the simplest one. Future studies using the LBA could corroborate if in fact the simplest model generalizes better than the model with variability in non-decision time.

We showed that drift rates estimated with the model are directly related to discounted subjective values independently derived from behavioral models of intertemporal choice. The drift rate parameters of the LBA model therefore have a direct psychological interpretation and suggest a powerful means to estimate subjective values independent of assuming and fitting a specific form for temporal discounting (e.g. the hyperbolic model in Equation 1). In contrast, we are uncertain about how to interpret the variability in non-decision times across value conditions. On average, non-decision times decreased with increased difficulty. Moreover, although median RT showed a modest relationship with non-decision times, median RT increased with choice difficulty, reflecting a dependence on accumulation rates. Non-decision times also correlated strongly with minimum RT, which did not vary systematically across value conditions, but was highly variable across subjects. This suggests that our best-fitting model is reflecting the fact that minimum RT varies considerably across value conditions. It is unclear what to conclude from these findings. Our belief is that non-decision times capture idiosyncratic differences in choice strategies and valuation processes across subjects and that incorporating a parameter to absorb these trends improves model fits overall and the interpretability of drift rates more specifically.

In summary, we have demonstrated that an LBA model provides an excellent description of the choice process in intertemporal decision making. The model fits RT distributions, provides an explanation for interdependence between RT and choice probability, and can be interpreted in terms of value accumulation. These results validate the LBA model as a complementary tool to temporal discounting models for studying the cognitive and neural mechanisms of intertemporal choice. Because the LBA has been applied to a wide range of perceptual decision making tasks, our findings not only demonstrate that a general mechanism of evidence accumulation drives decision making but also support a common and analytically tractable framework for explaining it.
